# Molecular interaction studies of Deguelin and its derivatives with Cyclin D1 and Cyclin E in cancer cell signaling pathway: The computational approach

**DOI:** 10.1038/s41598-018-38332-6

**Published:** 2019-02-11

**Authors:** Kiran Bharat Lokhande, Shuchi Nagar, K. Venkateswara Swamy

**Affiliations:** Bioinformatics Research Laboratory, Dr. D. Y. Patil Biotechnology and Bioinformatics Institute, Dr. D. Y. Patil Vidyapeeth, Pune, 411033 India

## Abstract

Deguelin is a major active ingredient and principal component in several plants and it is a potential molecule to target proteins of cancer cell signaling pathway. As a complex natural extract, deguelin interacts with various molecular targets to exert its anti-tumor properties at nanomolar level. It induces cell apoptosis by blocking anti-apoptotic pathways, while inhibiting tumor cell multiplication and malignant transformation through p27-cyclin-E-pRb-E2F1- cell cycle control and HIF-1alphaVEGF antiangiogenic pathways. *In silico* studies of deguelin and its derivatives is performed to explore interactions with Cyclin D1 and Cyclin E to understand the molecular insights of derivatives with the receptors. Deguelin and its derivatives are minimized by Avogadro to achieve stable conformation. All docking simulation are performed with AutoDockVina and virtual screening of docked ligands are carried out based on binding energy and number of hydrogen bonds. Molecular dynamics (MD) and Simulation of Cyclin D1 and Cyclin E1 is performed for 100 ns and stable conformation is obtained at 78 ns and 19 ns respectively. Ligands thus obtained from docking studies may be probable target to inhibit cancer cell signaling pathways.

## Introduction

In spite of extensive research on cancer and its cellular pathways, target identification and drug development, cancer still remains the major cause of death in economically developing and developed countries. Progression through the cell cycle checkpoints is regulated by complex interactions of cyclin and cyclin-dependent kinases (CDKs). One such cell cycle pathway, is well studied and has been shown to be abnormal in large number of tumors^1,2^. The pRb/p16/cyclin D1 cell cycle control pathway as it is a part of CDK^3^. CDKs contain two subunits, one is catalytic Cdk subunit and another is regulatory cyclin subunit that activate Cdk. Each phase of the cell cycle has a unique profile of cyclin-Cdk activity. Two types of cyclin-Cdks regulate the transportation of mammalian cells from quiescence into S phase of cell cycle: the D-type cyclins, which activates Cdk4/6, and cyclin E, which activates Cdk2^4^.

Cyclin D1 is an important regulator of cell cycle progression and can function as a transcriptional co-regulator^5^. Cyclin D1 induction of cell migration is CDK-dependent function^6^. Amplification or rearrangement of cyclin D1 gene-located on the chromosome 11q13, as well as overexpression of cyclin D1 protein has been described in a wide spectrum of human cancers such as squamous cell carcinomas of head and neck, esophagus, tongue and larynx and carcinomas of uterine cervix, astrocytoma’s, non-small-cell lung cancers and soft tissue sarcomas^7^. Apart from cyclin D1, cyclin E is also extensively studied in many cancers like carcinomas (breast, lung cervix, endometrium, and GI tract), lymphoma, leukemia, sarcomas and adrenocortical tumors. Cyclin E-CDK2 catalytic activity is required to down-regulate p27 protein. Forced expression of p27 Kip1 in proliferating cells arrests the cell cycle^8^. CDK4 and CDK6, which is associated with cyclin D and CDK2 which associates with cyclin E, are rate limiting for progression through G1 and into S-phase of the vertebrate cell cycle^9^. In contrast, cyclin E-Cdk2 deregulation leads to development of cancer^4^.

Deguelin is a natural retinoid extracted from several plants species, including Derris trifoliata, Mundulea sericea and Tephrosin veogelii and has shown great potential as a cancer chemo-preventive and therapeutic agent for cancer^10–12^. Research indicates that deguelin on animal models of mice, rat and mouse has effectively reduced the incidence of chemically induced skin tumors, mammary tumors^13^, colonic aberrant crypt foci^14^ and pre-neoplastic lesion formation in mammary gland in organotypic culture^15^. Deguelin induces apoptosis in association with the down regulation of cyclin D1, p21, pRb and regulates the G1/S and G2/M checkpoint^16^. Cell cycle abnormalities are important feature of the procession of human cancers. Deguelin has been found to regulate cell cycle in colon cancer cells by stimulating p27 expression^17^. Cyclin D1 and cyclin E is dramatically downregulated with treatment of deguelin^18,19^.

Thus, in the light of the reports stated above, it is evident that deguelin has shown promising chemopreventive and therapeutic activities in diverse types of cancer. Our study shows that, interaction of deguelin and its derivatives with cyclin D1 and cyclin E, to understand molecular insights in to cell cycle arrest. The effectiveness of deguelin can be enhanced through designing its derivatives by applying advanced computational approaches like molecular modeling, docking, dynamics and simulation for initial screening of leads. Molecular Docking calculates the binding energy, which is crucial to interpret the biological activity of ligand molecules^20^. Molecular dynamic simulation (MDS) is a computer simulation technique, used to monitor and evaluate the physical movements of atoms and molecules^21^. MDS permitted us to measure flexibility, rigidity and secondary structure prediction in terms of gain or loss during the simulation time^22^. At different time step of simulation, conformational flexibility of a receptor alter its interaction with ligand^23^, because convergence of amino acid pattern^24^.

## Results and Discussion

### Virtual screening and energy minimization

PubChem database is searched to obtain compounds having structural similarity with deguelin. The search showed 181 compounds to have 95% similarity with deguelin. Deguelin and its 181 derivatives are energy minimized using Steepest Descent method and Universal Force Field (UFF) and all the minimized compounds are subjected to the docking calculations with cyclin D1 and cyclin E receptor.

### Sequence analysis

**Figure 1:**
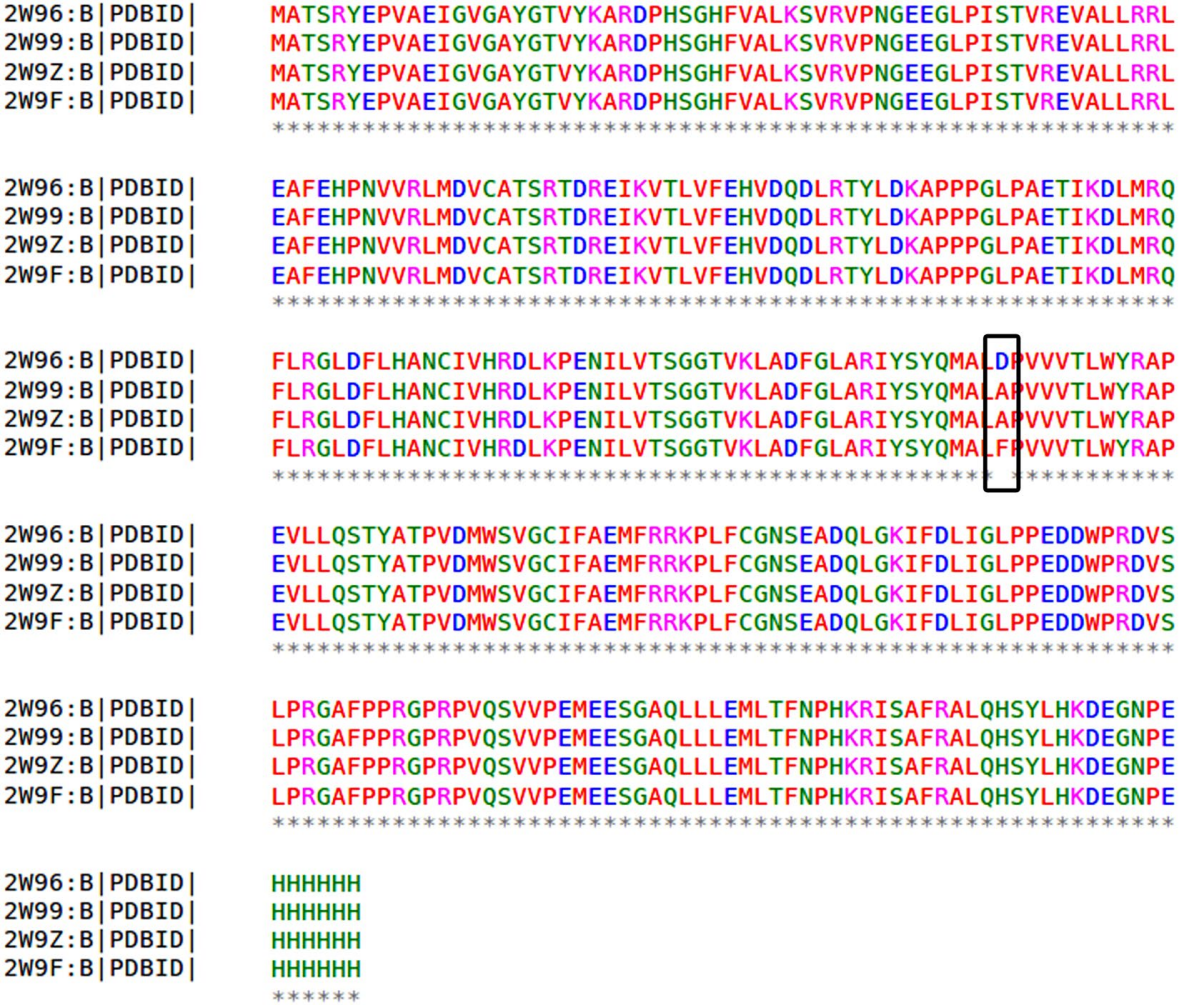
Multiple sequence alignment for four crystal structure of cyclin D1. Consensus residues are shown in the asterisk mark. The black box represents residue number 169 get mutated. Asp169Ala mutation found in the PDB ID: 2W96 and PDB ID: 2W9Z and also in the PDB ID: 2W9F Asp169Phe mutation occur.

Cyclin D1 crystal structure [PDB ID: 2W96 (Resolution: 2.30A^°^), 2W99 (Resolution: 2.80A^°^), 2W9Z (Resolution: 2.45A^°^) and 2W9F (Resolution: 2.85A^°^)] are downloaded and Multiple Sequence Alignment (MSA) is performed. It is observed that the inhibitory site residues are conserved in all the structures. The alignment shows that except for residue number 169, all others residues are identical (Fig. 1). Asp169Ala mutation is observed among three structures (2W96 to 2W99 and 2W9Z). Apart from existing structures another mutation absorbed at same position Asp169Phe in 2W96 and 2W9F.

**Figure 2:**
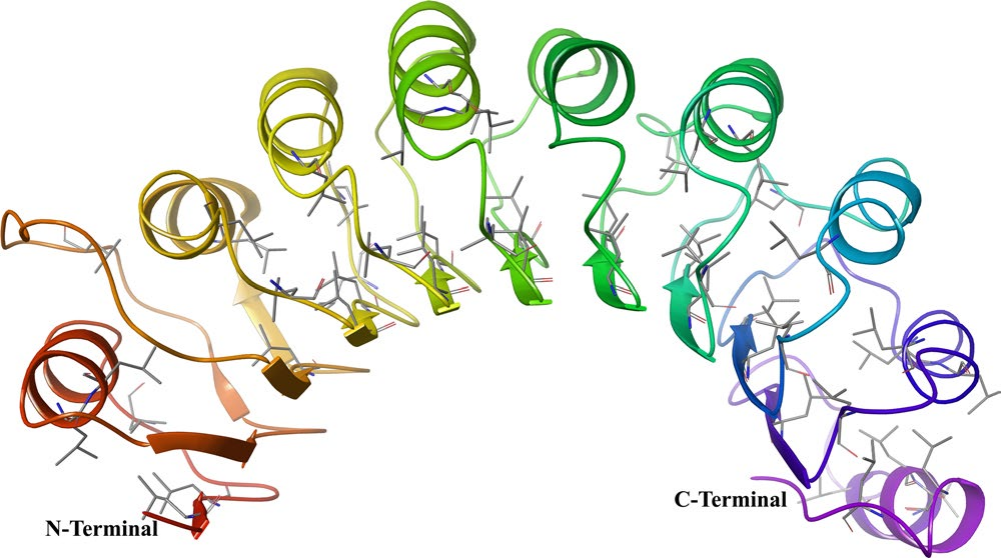
Leucine-rich repeats present in the cyclin E receptor (PDB ID: 2AST), that forms alpha/beta horseshoe fold.

Cyclin E [PDB ID: 2AST (Resolution: 2.30A^°^)], has motif called Leucine-rich repeats (LRR). These repeats responsible for formation of beta-alpha structural units (Fig. 2) fold in horseshoe (or arc) shape on the concave face. LRR are involved in protein-protein interaction as domain exposed to the solvent^25,26^. Two residues from inhibitory site present in this LRR domain including Phe2169 and Val2192.

### Structural analysis

**Figure 3:**
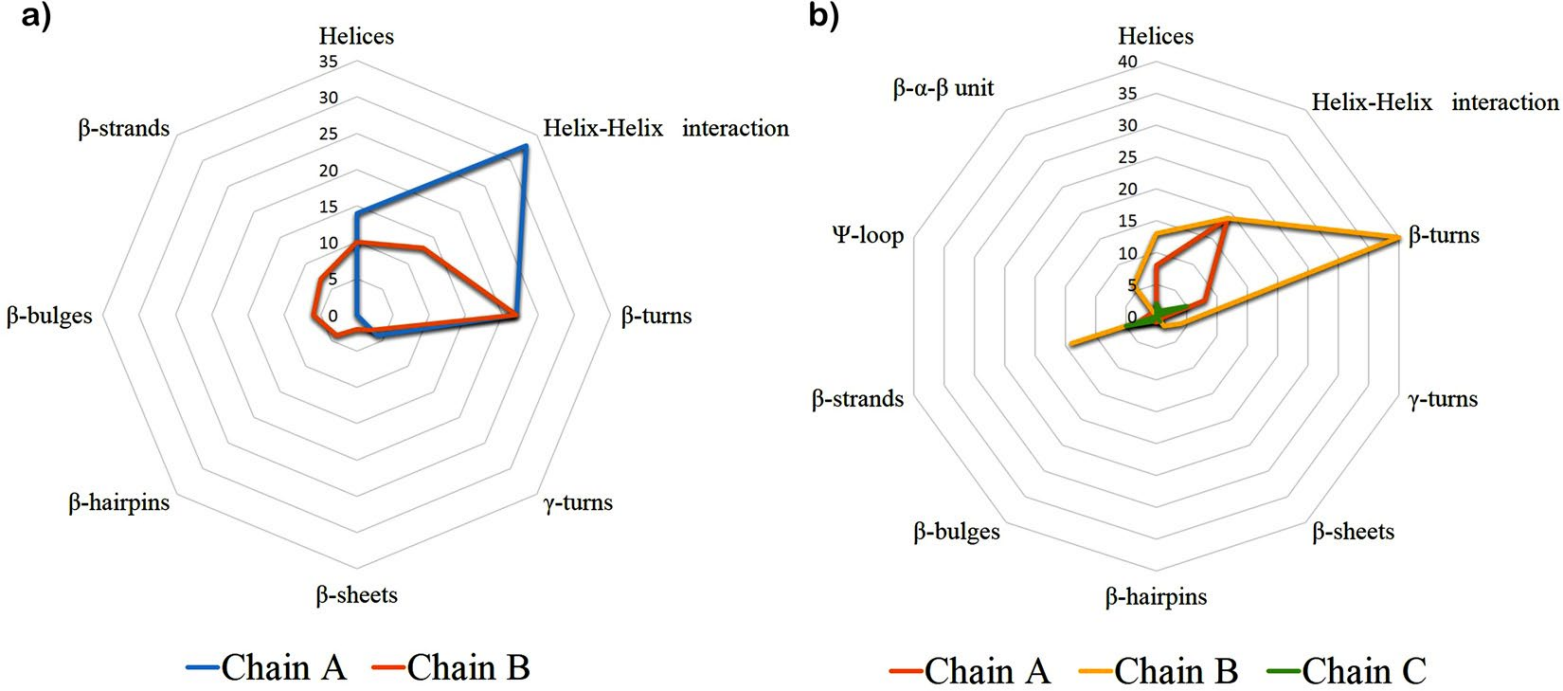
(**a**) Secondary structure prediction of cyclin D1 (PDB ID: 2W96). The secondary from the chain A presented in the cyan colour and from the chain B presented in the orange colour. (**b**) Secondary structure prediction of cyclin E (PDB ID: 2AST). The secondary from the chain A presented in the orange colour and from the chain B and chain C presented in the yellow and green colour respectively.

PROMOTIF^27^ program provides information about secondary structure of given protein for analysis. As per the program 2W96 contains chain A having 249 amino acids along with 14 helices, 33 helix-helix interactions, 22 beta-turns and 4 gamma-turns and chain B has 267 amino acids having secondary elements like, 2 sheets, 4 beta-hairpins, 6 beta-bulges, 7 strands, 10 helices, 13 helix-helix interaction, 22 beta-turns and 3 gamma-turns (Fig. 3(a)). At the inhibitory of cyclin D1 presents Ile12, Val20, Ala33, Val77, Phe93, Glu94 and Leu147 residues from chain B.

In case of 2AST, there are three chains, chain A, B and C. Among these chain C is small chain containing 69 amino acids. Chain A has 159 amino acids having 1 sheet, 1 beta-hairpins, 1 psi-loop, 1 beta-bulge, 3 strands, 8-helices, 19 helix-helix interactions, 8 beta-turns and 1 gamma-turns. The B chain of 2AST has 336 amino acids and it includes, 2 sheets, 6 beta-alpha- beta unit, 14 strands, 13 helices, 19 helix-helix interaction, 40 beta-turns and 4 gamma-turns. Chain C having 1 sheet, 2 beta-hairpins, 1 beta-bulge, 5 strands, 2-helices, 1 helix-helix interactions, 5 beta-turns (Fig. 3(b)). Inhibitory site residues for 2AST has Phe2169 and Val2192 which are present in beta-strands of chain B.

### MD simulation analysis

**Figure 4:**
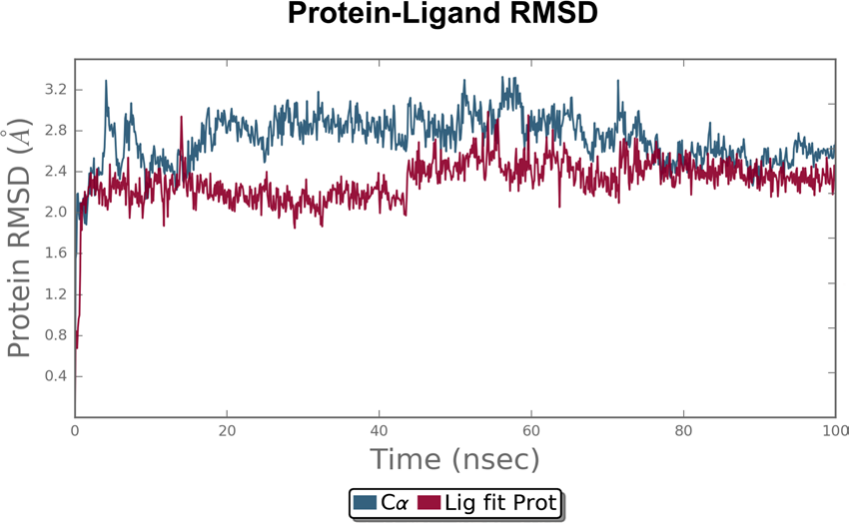
Root mean square deviation (RMSD) plot for cyclin D1-Deguelin complex during 100 ns of molecular dynamic simulation. Deguelin shown in the red colour and cyclin D1 in the black colour.

**Figure 5:**
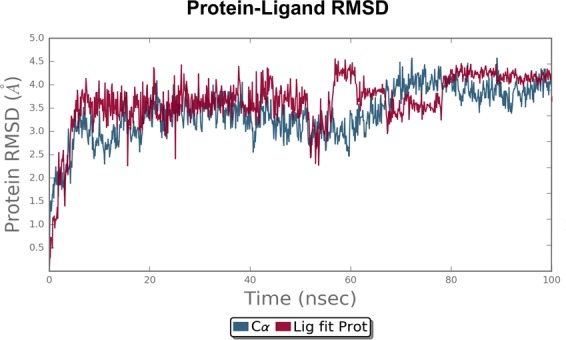
Root mean square deviation (RMSD) plot for cyclin E-Deguelin complex during 100 ns of molecular dynamic simulation. Deguelin shown in the red colour and cyclin E in the black colour.

To explore dynamic perturbation in the conformation of the protein-ligand complex, molecular dynamic simulation are carried out. Over the course of 100 ns simulation period for both receptor, the potential energy tends to decrease, which indicates the stabilization of the system. The conformation obtained during the 100 ns simulation for the receptor-ligand complex are analyzed. Root mean square deviation (RMSD) is calculated for the protein and ligand during the course of simulation trajectory of 100 ns, in order to calculate the average change in displacement of a selection of atoms for a particular frame with respect to a reference frame. The RMSD plot of the deguelin-cyclin D1 and deguelin-cyclin E complexes as shown in Figs 4 and 5, indicate that initially conformational fluctuations observed in the receptor complex system till 78 ns and 19 ns respectively, further stabilizes in production phase. The average RMSD of 2.4 A^°^, for cyclin D1 and RMSD of 3.5 A^°^ for cyclin E is obtained. Dynamically stable conformations of both receptors are used for the re-docking analysis to explore the stability of top selected ligands with both cyclin D1 and cyclin E receptor. Furthermore, to probe the flexibility of the deguelin and both receptors complexes, the root mean square fluctuation (RMSF) for C-alpha atoms of all the residues are compared with duration of 100 ns

**Figure 6:**
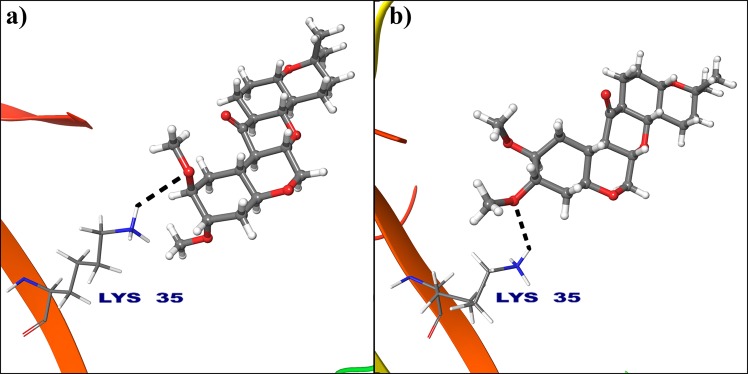
Deguelin shown in the binding pocket of cyclin D1. (**a**) Docked pose of deguelin in crystal structure of cyclin D1. **(b**) Docked pose of deguelin in stable conformation of cyclin D1 after 100 ns MD simulation.

MD simulations. It is notable that, the RMSF trajectories of cyclin D1 (Supplementary Fig. 1) is suggesting stability of the complexes with a related more rigid and stable conformations than cyclin E (Supplementary Fig. 2). The analysis reveals that, hydrogen atom of protonated −NH3+ group of Lys35 from 2W96 binds to oxygen atom from electron donating methoxy group of deguelin at 2nd position (Fig. 6(a)). But, this binding is completely changed in dynamically stable conformation due to orientation of deguelin during simulation and oxygen atom at 3rd position of deguelin interacted with protonated −NH3+ group of Lys35 (Fig. 6(b)). This conformation of deguelin exhibits strong interaction in stable conformation of cyclin D1.

**Figure 7:**
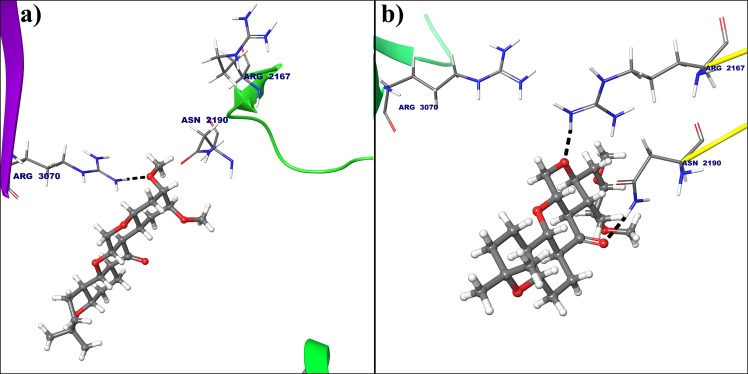
Deguelin shown in the binding pocket of cyclin E. **(a**) Docked pose of deguelin in crystal structure of cyclin E. (**b**) Docked pose of deguelin in stable conformation of cyclin E after 100 ns MD simulation.

In case of cyclin E hydrogen atom from amine group of Arg3070 forms hydrogen bond with oxygen atom from electron donating methoxy group of deguelin at 3rd position (Fig. 7(a)), but this interaction was not seen in dynamically stable conformation cyclin E receptor. Instead of that hydrogen atom from amine group of Arg2167 and hydrogen atom of acetamide from Asn2190 are interacted with oxygens at 7th and 8th of deguelin respectively because of deguelin is completely translate its orientation throughout MD simulation (Fig. 7(b)). MM-GBSA free energy calculations result in a more favorable total MM-GBSA binding energy for the deguelin with cyclin D1 (−77.79 kcal/mol) as compared to deguelin with cyclin E (−38.80 kcal/mol).

### Molecular docking study

To study the binding modes of the deguelin and its derivatives in cyclin D1 and cyclin E, initially we performed the molecular docking study of deguelin and its derivatives with cyclin D1 and cyclin E using AutoDock Vina 1.1.2. After running docking calculations, nine conformers are generated for each and all the conformers ranked in log file based on their docking score. 24 best docked score conformer is selected for cyclin D1 and 5 for Cyclin E, and speculated concerning the detailed information about binding mode in inhibitory site cavity. The best 3 scoring ligands common in cyclin D1 (Tables 1 and 2) and for cyclin E (Tables 3 and 4) are selected for analysis. For the ranking of ligands from molecular docking, binding affinity and bond distances along with orientation is considered. On the basis of docking score it can be concluded that compounds namely Deg-32, Deg-40 and Deg-49 are more effective against cyclin D1 and cyclin E as compared to deguelin, also its binding affinity is more than deguelin. These deguelin derivatives could be useful for identification and development of new therapeutic agents against cancer.

**Table 1:** Binding affinity of Deguelin and its derivatives with crystal structure of cyclin D1 receptor.

Sr. No.	Compound Name	PubChem CID	Binding Affinity (kcal/mol)	Interacting Residues	Bond Type	Bond Distance(A^0^)
1	Deguelin	107935	−8.9	Lys35	H-Bond	2.74
2	Deg-32	89254848	−10.2	Lys35 Val96 Asp97 Asp158	H-Bond H-Bond 2H-Bond H-Bond	2.00 2.08 1.98 and 2.32 1.61
3	Deg-40	71457423	−10.1	His95	H-Bond	2.16
4	Deg-49	68251256	−9.4	Ala96	H-Bond	1.93

**Table 2:** Binding affinity of Deguelin and its derivatives with dynamically stable conformation of cyclin D1 receptor.

Sr. No.	Compound Name	PubChem CID	Binding Affinity (kcal/mol)	Interacting Residues	Bond Type	Bond Distance (A^0^)
1	Deguelin	107935	−8.7	Lys35	H-Bond	2.35
2	Deg-32	89254848	−10.9	Gly13 His95 Glu144	H-Bond H-Bond H-Bond	2.14 2.23 1.64
3	Deg-40	71457423	−9.3	Gly13 Val14	H-Bond H-Bond	2.07 1.67
4	Deg-49	68251256	−10.1	Gly13	H-Bond	1.74

**Table 3:** Binding affinity of Deguelin and its derivatives with crystal structure of cyclin E receptor.

Sr. No.	Compound Name	PubChem CID	Binding Affinity (kcal/mol)	Interacting Residues	Bond Type	Bond Distance (A^0^)
1	Deguelin	107935	−7.8	Arg3070	H-Bond	1.75
2	Deg-32	89254848	−8.8	Asn2190 Ser2191 Ile2193 Arg3070	H-Bond H-Bond H-Bond 2H-Bond	2.36 2.05 1.83 1.81 and 2.38
3	Deg-40	71457423	−8.4	Ile2193 Arg2217 Arg3070	H-Bond 2H-Bond 2H-Bond	2.30 1.97 and 2.30 1.72 and 2.35
4	Deg-49	68251256	−8.8	Arg3070	2H-Bond	1.58 and 1.80

**Table 4:** Binding affinity of Deguelin and its derivatives with dynamically stable conformation of cyclin E receptor.

Sr. No.	Compound Name	PubChem CID	Binding Affinity (kcal/mol)	Interacting Residues	Bond Type	Bond Distance (A^0^)
1	Deguelin	107935	−8.3	Arg2167 Asn2190	H-Bond H-Bond	1.89 1.85
2	Deg-32	89254848	−9.1	Arg2167 Asn2190 His3056	3-Bond H-Bond H-Bond	1.88, 1.86 and 2.02 1.78 1.73
3	Deg-40	71457423	−8.6	Arg2167 Leu3037 Arg3070	H-Bond H-Bond H-Bond	1.99 2.36 1.69
4	Deg-49	68251256	−8.8	Arg2167 Arg3070	H-Bond H-Bond	1.94 1.84

**Figure 8:**
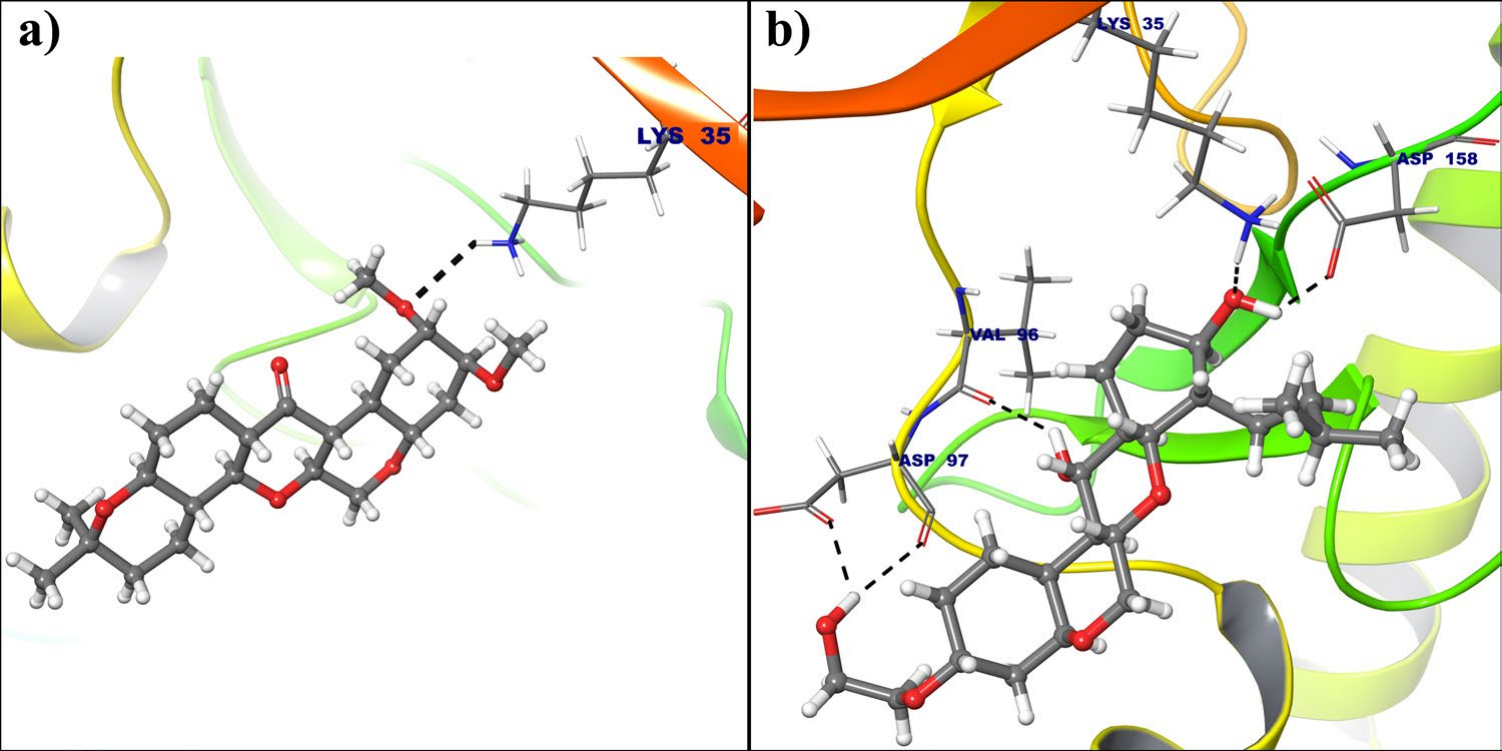
(**a**) Binding mode of Deguelin with the crystal structure of cyclin D1. (**b**) Binding mode of Deg-32 with the crystal structure of cyclin D1. Dashed black lines shows hydrogen bonds.

**Figure 9:**
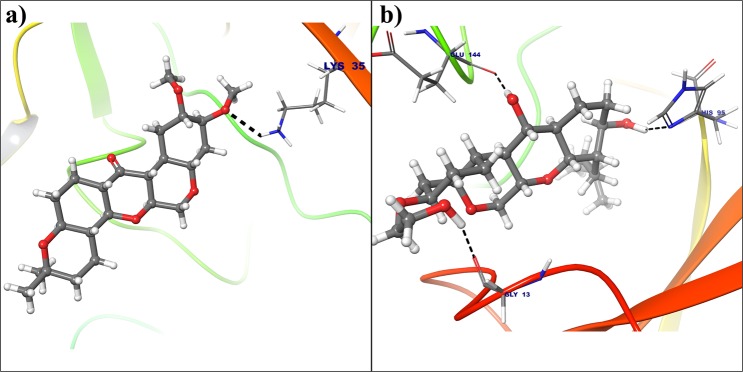
(**a**) Binding mode of Deguelin with the dynamically stable confirmation of cyclin D1. (**b**) Binding mode of Deg-32 with the dynamically stable confirmation of cyclin D1. Dashed black lines shows hydrogen bonds.

**Figure 10:**
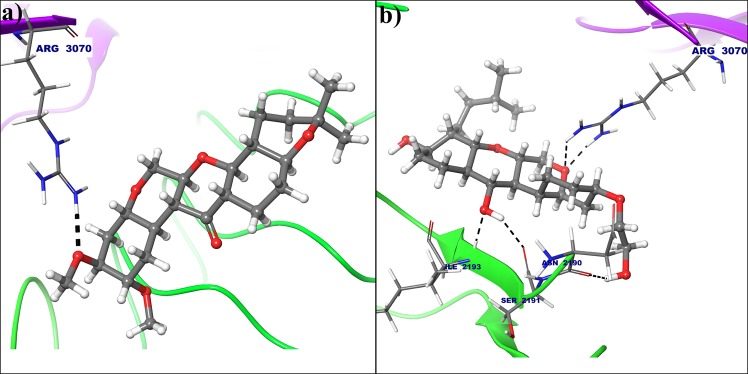
(**a**) Binding mode of Deguelin with the crystal structure of cyclin E. (**b**) Binding mode of Deg-32 with the crystal structure of cyclin E. Dashed black lines shows hydrogen bonds.

**Figure 11:**
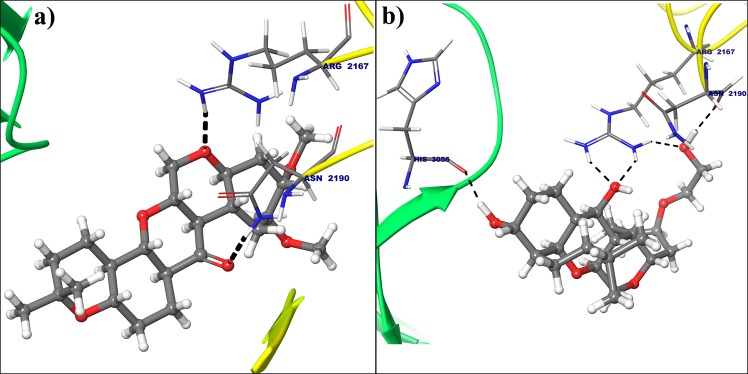
(**a**) Binding mode of Deguelin with the dynamically stable confirmation of cyclin E. (**b**) Binding mode of Deg-32 with the dynamically stable confirmation of cyclin E. Dashed black lines shows hydrogen bonds.

All the selected compounds are re-docked with dynamically stable cyclin D1 and cyclin E obtained after 100 ns molecular simulation. By analyzing the result obtained from docking calculation both for crystal structure and dynamically stable conformation, it can be said that the compound Deg-32 can behave as a lead compound for cyclin D1. The compound Deg-32 is having high score in both conformation with binding energy −10.2 kcal/mol (Fig. 8(b)) and −10.9 kcal/mol (Fig. 9(b)) for crystal structure and dynamically stabilized structure respectively. Deguelin having binding energy −8.9 kcal/mol for crystal structure of cyclin D1 (Fig. 8(a)) and −8.7 kcal/mol for dynamically stabilized structure (Fig. 9(a)), indicating that Deg-32 shows better binding affinity than deguelin for cyclin D1. Deg-32 is found to be more stable in the inhibitory site of cyclin E receptor (crystal and dynamically stable) having binding energy −8.8 kcal/mol (Fig. 10(b)) and −9.1 kcal/mol (Fig. 11(b)) respectively. It is also noted that deguelin interacts with cyclin E with binding energy −7.8 kcal/mol (Fig. 10(a)) and −8.3 kcal/mol (Fig. 11(a)), that suggests Deg-32 to be more active than deguelin for cyclin E.

**Figure 12:**
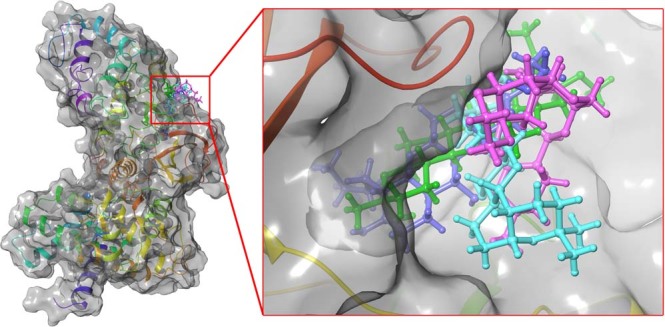
Superposition of the docking poses of Deguelin and its top 3 scored derivative in the inhibitory site of dynamically stable confirmation of cyclin D1. The structure of Deguelin presented in the blue colour, while structure of Deg-32 presented in the green colour, Deg-40 in pink and Deg-49 in cyan colour.

**Figure 13:**
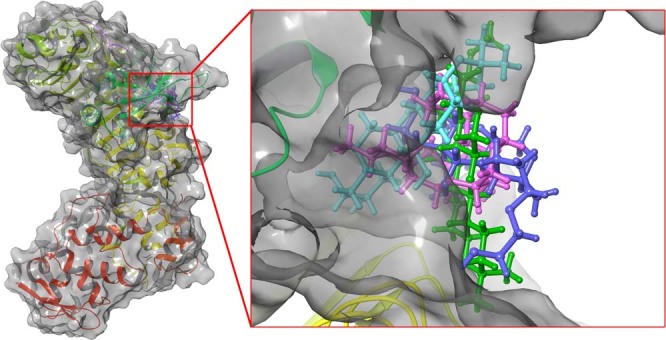
Superposition of the docking poses of docked Deguelin and its top 3 scored derivative in the inhibitory site of dynamically stable confirmation of cyclin E. The structure of Deguelin presented in the blue colour, while structure of Deg-32 presented in the green colour, Deg-40 in pink and Deg-49 in cyan colour.

**Figure 14:**
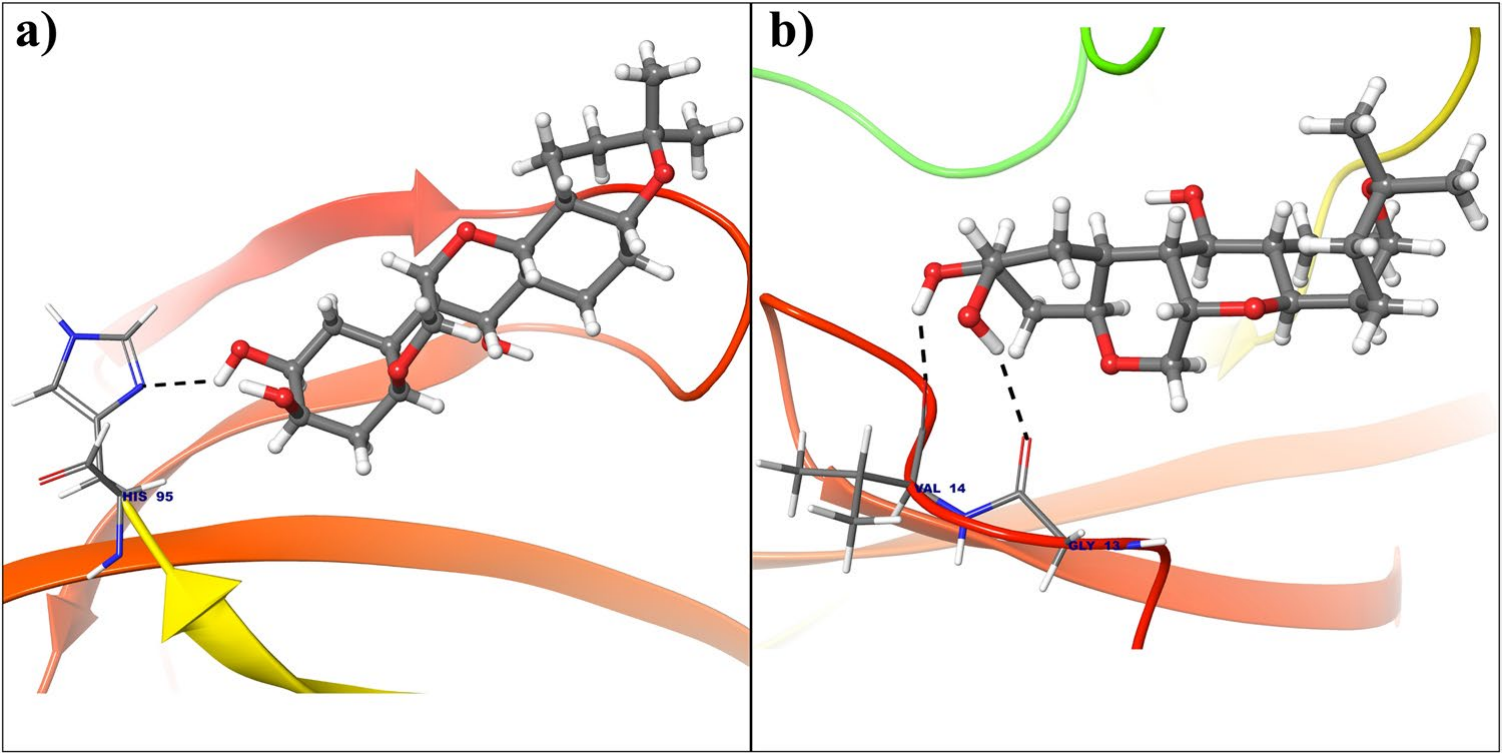
(**a**) Binding mode of Deg-40 with the crystal structure of cyclin D1. (**b**) Binding mode of Deg-40 with dynamically stable confirmation of cyclin D1. Dashed black lines shows hydrogen bonds.

**Figure 15:**
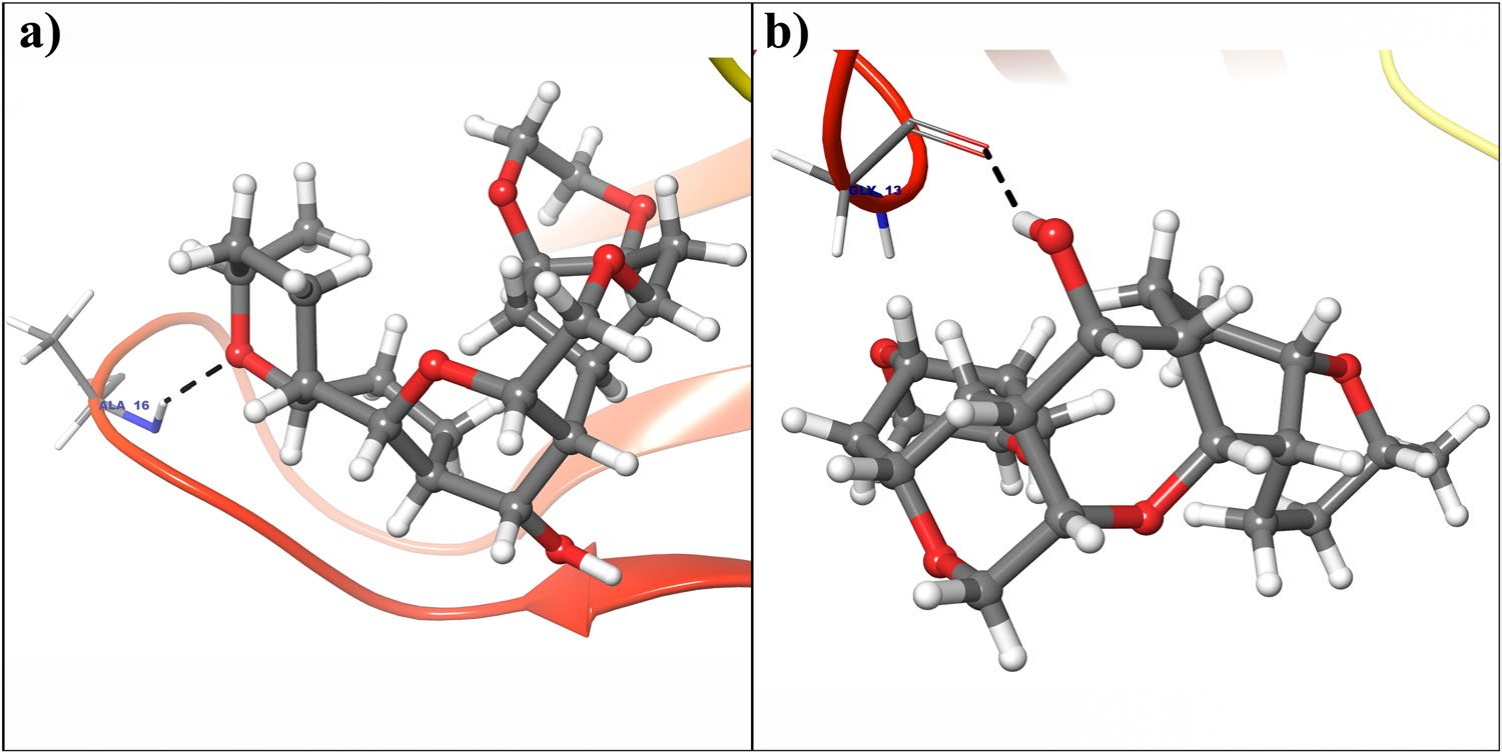
(**a**) Binding mode of Deg-49 with the crystal structure of cyclin D1. (**b**) Binding mode of Deg-49 with dynamically stable confirmation of cyclin D1. Dashed black lines shows hydrogen bonds.

Considering top 3 scored ligands along with deguelin in the inhibitory site of cyclin D1 and cyclin E are presented in the Figs 12 and 13 respectively. Docking analysis reveals that top ranked deguelin derivative Deg-32 forms H-bond interaction with Lys35, Val96 and Asp158 having a bond length of 2.0 A^°^, 2.08 A^°^ and 1.61 A^°^ respectively. Another residue Asp97 interacts with deguelin forming two hydrogen bond with bond length of 1.98 A^°^ and 2.32 A^°^ in crystal structure of cyclin D1, while in dynamically stable conformation of same receptor, Deg-32 forms three H-bond interaction with Gly13, His95 and Glu144 having bond distance of 2.14 A^°^, 2.23 A^°^ and 1.64 A^°^ respectively. Deguelin also shows H-bond interaction with Lys35 bond distance in crystal structure of cyclin D1 but 3.5 A^°^ bond distance found in stable conformation of MD simulation. Deg-40 and Deg-49 also forms stable complex with cyclin D1. Deg-40 forms hydrogen bonding with His95 of crystal structure of cyclin D1 having −10.1 kcal/mol binding energy (Fig. 14(a)). Residues like Gly13 and Val14 from dynamically stabilized conformation of cyclin D1, makes hydrogen bonding with Deg-40 having −9.3 kcal/mol binding energy (Fig. 14(b)). Ala16 from crystal structure of Cyclin D1 and Gly13 from stable conformation of cyclin D1 forms hydrogen bonding with Deg-49 having −9.4 kcal/mol and −10.1 kcal/mol binding energy respectively (Fig. 15(a,b)).

In case of cyclin E receptor, Deg-32 forms H-bond interactions with Asn2190, Ser2191, Ile2193 residues having 2.36 A°_,_ 2.05 A^°^ and 1.83 A^°^ bond distance respectively. Arg3070 shows two hydrogen bonding with Deg-32 showing bond distances of 1.81 A^°^ and 2.38 A^°^ respectively. The dynamically stabilized conformation shows that Deg-32 forms two H-bond interactions with Asn2190 and His3056 having bond distance of 1.78 A^°^ and 1.73 A^°^ respectively. Arg3070 makes three hydrogen bonding with Deg-32 having 1.88 A^°^, 2.76 A^°^ and 1.82 A^°^ bond distances. Also deguelin shows H-bond interaction Arg3070 with 1.75 A^°^ bond distance in crystal structure of cyclin E and H-bond interactions with residue Arg2167 with 1.89 A^°^ bond distance and Asn2190 with 1.85 A^°^ bond distance. Another residue Arg3070 form hydrogen bond with 1.82 A^°^ distance with Deg-32 in stable conformation obtained from MD simulation.

**Figure 16:**
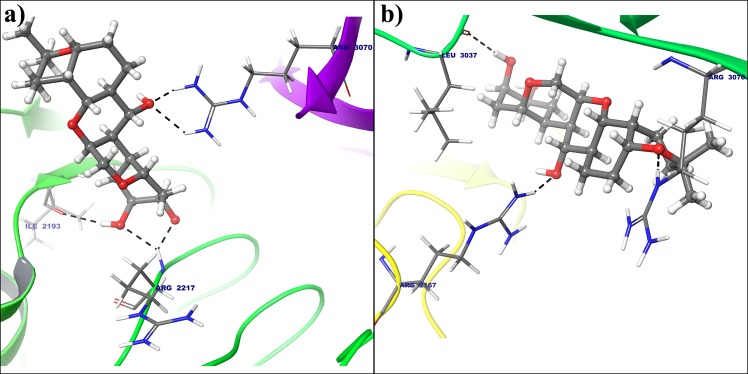
(**a**) Binding mode of Deg-40 with the crystal structure of cyclin E. (**b**) Binding mode of Deg-40 with dynamically stable confirmation of cyclin E. Dashed black lines shows hydrogen bonds.

**Figure 17:**
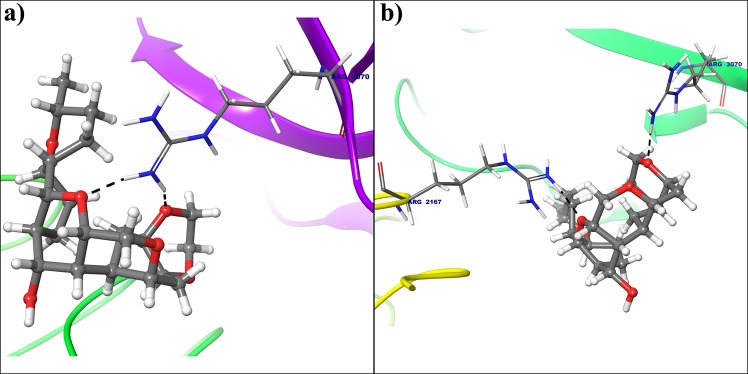
(**a**) Binding mode of Deg-49 with the crystal structure of cyclin E. (**b**) Binding mode of Deg-49 with dynamically stable confirmation of cyclin E. Dashed black lines shows hydrogen bonds.

Five hydrogen bond formation occurred from Deg-40 with crystal structure of cyclin E (Fig. 16(a)). In that residue Ile2193 form one H-bonding, Arg2217 and Arg3070 shows two hydrogen bonds each with Deg-40 having −8.4 kcal/mol binding energy. Arg2167, Leu3037 and Arg3070 from dynamically stable conformation of cyclin E forms hydrogen bonding with Deg-40 with −8.6 kcal/mol binding energy (Fig. 16(b)). Another lead molecule Deg-49 makes two hydrogen bond interaction with Arg3070 in crystal structure (Fig. 17(a)) and two hydrogen bond interaction with Arg2167 and Arg3070 from stable conformation of cyclin E receptor(Fig. 17(b)) having −8.8 kcal/mol binding energy for both the receptor.

## Conclusion

The present research describes the result of screening and identification of potent anti-cancerous deguelin and its derivatives, targeted towards a key receptors namely cyclin D1 and cyclin E in cell signaling pathway. Screening of deguelin derivatives from PubChem database revealed 181 derivatives through molecular property filter. The potency and efficacy of the successfully screened deguelin derivatives were further analyzed by performing molecular docking analysis. It was noticed that 24 derivatives were docked successfully with cyclin D1 and 5 derivatives with cyclin E receptor. Among them Deg-32, Deg-40 and Deg-49 were ranked higher as compared to deguelin as the most potent inhibitor. The calculated binding free energy of deguelin is notably weaker towards cyclin E receptor than cyclin D1. The result of MD simulation revealed least RMSD values for cyclin D1 and cyclin E as a stable conformation at solvent system and the docking validation was done after simulation with dynamically stable conformation suggesting that Deg-32 shows more affinity towards stable conformation of cyclin D1 and cyclin E. Thus, the present work makes a foundation for the development of Deg-32 as a potent anti-cancerous drug acting to inhibition of cyclin D1 and cyclin E.

## Methods

### Virtual Screening and energy minimization

Deguelin (CID: 107935), is used as a reference compound to perform virtual screening to search for deguelin analogues in PubChem database (http://pubchem.ncbi.nlm.nih.gov/search/search.cgi)^28^, with similarity threshold over 95% according to the Tanimoto’s coefficients^29,30^ and Lipinski rule of five^31^.

Screened structures are energy minimized using Avogadro software^32^, until atomic/angle stable conformation is obtained. Energy minimization is a procedure for searching a minimum on the potential energy surface starting from a higher energy initial structure. Avogadro allows building of chemical structure, visualization and analysis of molecule, structure optimization, quantum mechanical calculations and electron density calculations.

### Sequence retrieval and analysis

Four homologous of cyclin D1 and three homologous of cyclin E receptor sequences, from Homo sapiens (Human), are obtained from UniProtKB^33,34^ (www.uniprot.org). The multiple sequence alignment for each of cyclins (cyclin D1 and cyclin E), is performed by Clustal Omega^35^ (www.ebi.ac.uk/Tools/msa/clustalo/), online server. The crystal structure for cyclin D1 (PDB ID: 2W96) and cyclin E (PDB ID: 2AST) are retrieved from PDB database^36^ (www.rcsb.org/pdb/home.do). PDBsum^37^ is used for identifying pictorial summary of the key information on each macromolecular structure of both the proteins.

### Preparation of cyclin D1 and cyclin E for docking

The X-ray crystal structure of the cyclin D1 (PDB ID: 2W96) and cyclin E (PDB ID: 2WAST) receptors with 2.30 A^°^ resolution each is downloaded from Protein Data bank. Before performing docking calculation, all the water molecules are removed from the crystal structures, all hydrogen atoms and Gasteiger charges are added to every atom of the protein, rotatable bonds are selected using AutoDockTools-1.5.6^38^. The amino acid residues involved in binding interactions are Ile12, Val20, Ala33, Val77, Phe93, Glu94, His95, Val96, Gln98, Asp99, Thr102, Glu144, Leu147, Ala157 and Asp158 in the B chain of the protein. These residues are known to exist at ATP binding pocket^39^. For the cyclin E, inhibitory site residues are selected from the Server that give the information about cavity (www.scfbio-iitd.res.in/dock/ActiveSite.jsp) which are Phe2169, Val2192, Met3058, and Arg3070, which are similar to the site information from the cyclin E (PDB ID: 2AST)^40^. The grid box is generated for cyclin D1 and cyclin E, auto grid covered grid map dimensions 48 × 50 × 50 A^°^ with grid spacing of 0.375 A^°^.

### Molecular docking studies

For the molecular docking studies, crystal structure of cyclin D1 and cyclin E (PDB ID: 2AST) were considered. The grid box is generated based on the inhibitory site residue used for the docking calculation. Deguelin and its 181 derivatives (Deg-1 to Deg-181) are selected for docking using AutoDockTools-1.5.6 and converted in to pdbqt format, which stores the atomic coordinates, partial charges and AutoDock atom types. *In silico* screening and docking analysis of deguelin and 181 derivatives on the cyclin D1 and cyclin E receptor is carried out using AutoDock Vina 1.1.2^41^. AutoDock Vina, program for molecular docking as well as virtual screening and it automatically calculates the grids maps and cluster the result. The scoring function Vinardo (Vina RaDii Optimized) implemented for docking calculation due to that is a combination of Gaussian attraction and a quadratic repulsion^42^. Molecular Docking interactions are analyzed using Maestro (Schrodinger). Based on the docking score, the best conformation of each ligands is taken for further analysis.

### Molecular Dynamics Simulation and docking validation

Stability of docked complexes and interaction of cyclin D1 and cyclin E with the deguelin compound are simulated till 100 ns of molecular dynamics (MD) simulations using Desmond^43^. Using system builder of Desmond in the Maestro^44^ program, system for cyclin D1 and cyclin E immersed in a water filled cubic box of 1 A^°^ spacing containing 9837 and 20734 water molecules using extended simple point charge (SPC), a three point water model with periodic boundary conditions. The total charge of the solvent system were neutralized by adding Sodium (NA) ion. 6 NA of 11.09 mM molar concentration and 7 NA of 6.13 mM concentration ions added to the cyclin D1 and cyclin E respectively. The both systems are heated linearly at constant volume (NVT ensemble, 1 bar) from 0 to 300 K over 400 ps. Equilibration is obtained using Desmond protocol at constant pressure and temperature (NPT ensemble, 300 K, 1 bar) and the Berendsen coupling scheme with one temperature group. All bond length of hydrogen atoms are constrained using M-SHAKE. Cut off for Van Der Waals and short-range electrostatics interactions is kept at 10 A^°^. Long-range electrostatic interactions are computed using particle mesh Ewald (PME) summation. After stability, both of the systems are subjected to 100 ns MD simulation at 300 K temperature along with 1 bar pressure without restraints. The equations of motion are integrated by applying the leap-frog algorithm with a time step of 2 fs. After the system gets equilibrated, the stable conformation trajectories are captured for docking validation. All selected compounds from docking calculation based upon its binding energy are docked in both stable conformation obtained from trajectory for cyclin D1 and cyclin E, to explore the interaction stability. The conformational change of C-alpha backbone of cyclin D1 and cyclin E is been compared with initial conformations.

Molecular Mechanics Generalized Born Surface Area (MM-GBSA) method was used for the calculation of binding free energies between the deguelin and respective cyclin D1 as well as cyclin E receptor. As the MM-GBSA binding energies are approximate free energies of binding, a more negative value indicates stronger binding. The equilibrated trajectories from MDS was used in the MMGBSA calculations using Prime^45^ module. The VSGB solvent model was used for solvation model and the force field OPLS-2005 was used to parameterize the targets and the deguelin.

## Supplementary information

Supplementary File
