# Supplementary material for: Molecular interaction studies of Deguelin and its derivatives with Cyclin D1 and Cyclin E in cancer cell signaling pathway: The computational approach
Source: Sci Rep. 2019 Feb 11;9:1778. doi: 10.1038/s41598-018-38332-6 (PMC6370771; doi:10.1038/s41598-018-38332-6)
Supplement: Supplementary file 1 — Supplementary File [file 41598_2018_38332_MOESM1_ESM.pdf]

Molecular interaction studies of Deguelin and its derivatives with Cyclin D1 and Cyclin E in cancer cell signaling pathway: The computational approach

Kiran Bharat Lokhande[1], Shuchi Nagar[2], and K. Venkateswara Swamy[3*]

[1,2,3] Bioinformatics Research Laboratory, Dr. D. Y. Patil Biotechnology and Bioinformatics Institute, Dr. D. Y. Patil Vidyapeeth, Pune, 411033, India.

*venkateswara.swamy@gmail.com

Supplementary Figures:

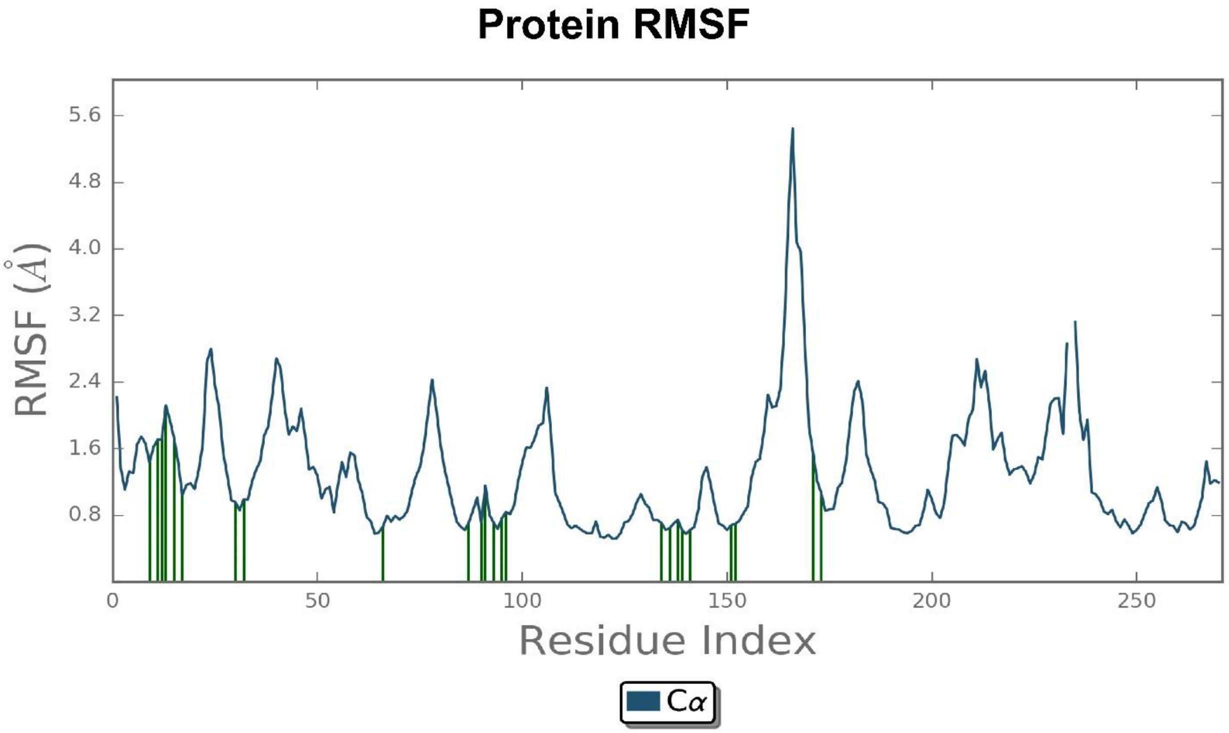

Supplementary Figure 1. Root mean square fluctuation (RMSF) plot for cyclin D1 during 100ns of molecular dynamic simulation. Protein residues that interact with the ligand are marked with green-colored vertical bars.

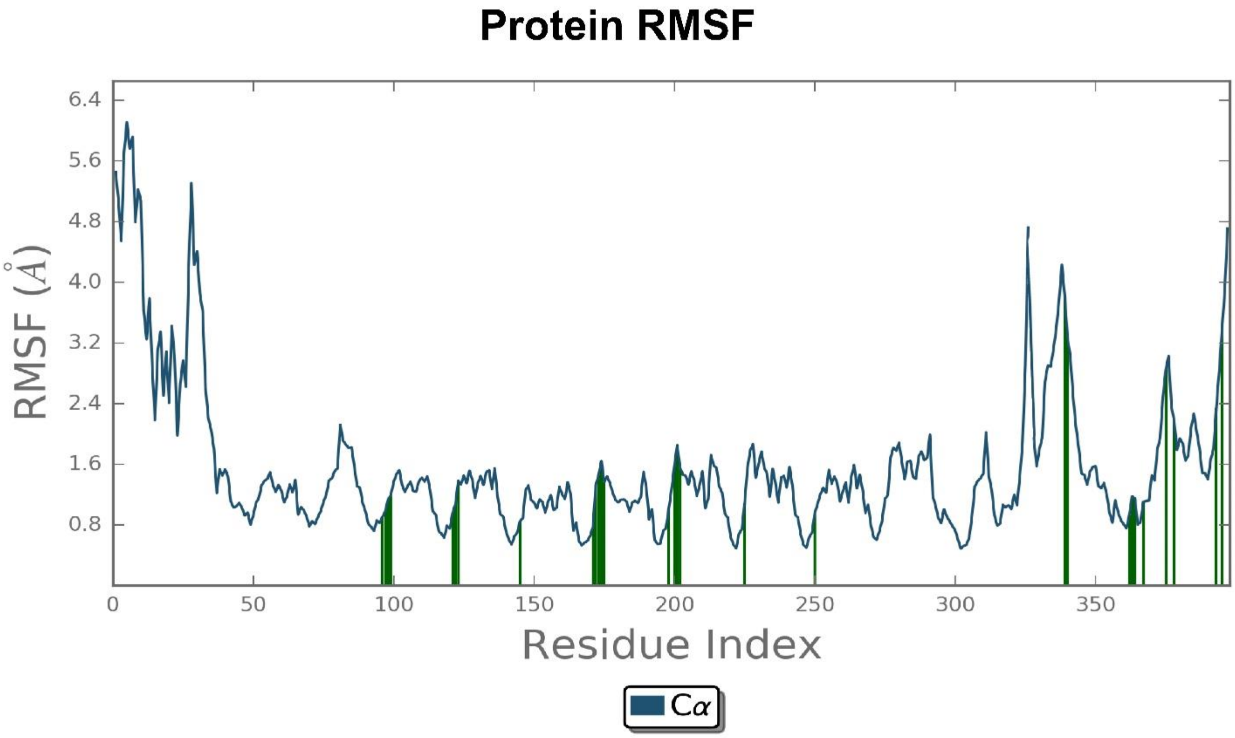

Supplementary Figure 2. Root mean square fluctuation (RMSF) for cyclin E during 100ns of molecular dynamic simulation. Protein residues that interact with the ligand are marked with green-colored vertical bars.
